# Development of a high-sensitivity vertical flow immunoassay for the detection of Rift Valley fever virus

**DOI:** 10.1128/spectrum.00341-25

**Published:** 2025-06-09

**Authors:** Alexander J. Summers, Haydon J. Hill, Jasmine P. Devadhasan, Jian Gu, Vanessa Berner, Sujata G. Pandit, Marcellene A. Gates-Hollingsworth, Kathryn J. Pflughoeft, Douglas C. Montgomery, Supriya Atta, Tuan Vo-Dinh, David P. AuCoin, Frederic Zenhausern

**Affiliations:** 1 Center for Applied NanoBioscience and Medicine, College of Medicine, University of Arizona 42283 , Phoenix, Arizona, USA; 2 Department of Microbiology and Immunology, University of Nevada Reno School of Medicine 12290, Reno, Nevada, USA; 3 Department of Basic Medical Sciences, The University of Arizona, College of Medicine 42283 , Phoenix, Arizona, USA; 4 School of Computing and Augmented Intelligence, Arizona State University 7864, Tempe, Arizona, USA; 5 Fitzpatrick Institute for Photonics, Departments of Biomedical Engineering, Duke University 3065, Durham, North Carolina, USA; 6 Honor Health Research Institute, Scottsdale, Arizona, USA; National Microbiology Laboratory, Winnipeg, Manitoba, Canada

**Keywords:** Rift Valley fever virus, vertical flow device, biothreat agents, colorimetric detection, rapid antigen test

## Abstract

Rift Valley fever (RVF) is a vector-borne, zoonotic infectious disease with a proven history of morbidity and mortality in both humans and animals. Rift Valley fever virus (RVFV) is categorized as a high-priority biothreat agent by the Centers for Disease Control and Prevention and poses a serious national threat due to its ease of dissemination and potential for social disruption. RVF often presents as a febrile disease without specific symptoms, making early-stage detection particularly challenging. As such, it is critical that rapid, sensitive, and specific diagnostics are available for the detection of RVFV. While lateral flow immunoassays (LFIs) have been developed and validated for point-of-care (POC) diagnostics, vertical flow immunoassays (VFIs) provide enhanced analytical sensitivity and are equally suitable for POC use. In this study, we developed a VFI system for the detection of RVFV, achieving a limit of detection of 0.78 ng/mL, which is a 2.5-fold increase in analytical sensitivity compared to an LFI prototype. Furthermore, minimal cross-reactivity was demonstrated when performing the assay with target analytes of other high-priority biothreats and one other common viral nucleoprotein. This high-sensitivity VFI has the potential to prove useful for the detection of RVFV and other high-priority biothreat agents at the POC.

**IMPORTANCE:**

In this study, we have developed a rapid, sensitive vertical flow immunoassay (VFI) for the detection of Rift Valley fever virus (RVFV) in spiked human serum. The prototype diagnostic described in this research was shown to be more sensitive than traditional methods, such as lateral flow dipstick tests. Moreover, the VFI is readily deployable at the point of care in resource-limited settings. The ability of the described diagnostic to accurately and rapidly detect RVFV in samples could expedite the delivery of life-saving care and thus improve patient outcomes.

## INTRODUCTION

Rift Valley fever virus (RVFV), a phlebovirus of the *Bunyaviridae* family, is a negative-sense RNA virus responsible for Rift Valley fever (RVF) (1). First discovered in the 1930s in Kenya, this mosquito-borne, zoonotic infectious disease is endemic to sub-Saharan Africa and the Arabian Peninsula (2, 3). RVFV causes a hemorrhagic fever with demonstrated morbidity and mortality in both livestock and humans (4). Humans can contract RVFV after being bitten by infected mosquitoes or following direct contact with the blood, bodily fluid, or tissue of an infected animal, or exposure to infectious aerosols generated during necropsy (2, 5). In humans, RVF often presents as a febrile disease with non-specific symptoms, making early-stage detection challenging. An overall case fatality rate (CFR) of 0.5%–3% has been reported for RVF; however, CFR can be as high as 50% in cases of severe disease (5–7). Currently, there is no licensed RVFV vaccine for humans, and healthcare practitioners remain without effective treatments, adequate diagnostics, or surveillance tools (8). The United States Centers for Disease Control and Prevention (CDC) lists RVFV as a Select Agent, acknowledging the pathogen as a possible biothreat and a risk to national security (https://www.selectagents.gov/sat/list.htm). The World Health Organization (WHO) also identified RVFV as a Blueprint priority disease due to the potential danger the virus poses to public health (https://www.who.int/activities/prioritizing-diseases-for-research-and-development-in-emergency-contexts). As such, it is imperative that rapid, sensitive, and specific point-of-care (POC) diagnostics are available for the detection, surveillance, and effective management of RVFV.

Current diagnostic methods for RVFV detection include nucleic acid amplification tests (NAATs), enzyme-linked immunosorbent assay (ELISA), lateral flow immunoassay (LFI), and electrochemical biosensors (9–17). Both NAATs and ELISAs require dedicated laboratory infrastructure, specialized equipment, and trained personnel. Furthermore, these assays can take days to provide results, making them inaccessible in many endemic regions. To date, commercial products exist exclusively for NAAT and ELISA techniques. LFIs have the benefit of being cost-effective, easy to use, and administrable at the POC. Prior reported LFIs for RVF were primarily serological tests, which were not suitable for early detection (13–15). To facilitate early detection, Cêtre-Sossah et al. developed and validated an LFI test targeting the RVFV nucleoprotein (NP), demonstrating the proof-of-concept of a rapid immunoassay (16). Electrochemical detection of RVFV NP has also been reported (17). Generally, low detection limits are advantageous in enabling detection of infections at early stages (18, 19). Recently, vertical flow immunoassays (VFIs) have demonstrated the ability to handle large sample volumes while maintaining high sensitivity and are suitable for use at the POC. Our team has developed both sensitive LFI and VFI devices with biothreat detection capabilities (20–25). The VFI prototypes have demonstrated up to 80× higher sensitivity than their LFI counterparts, including the capability of being readily multiplexed (26). However, a VFI has not been developed for the diagnosis of RVF.

This study addressed the need for a sensitive POC diagnostic for the detection of RVFV by developing a VFI test targeting RVFV NP. First, monoclonal antibodies (mAbs) for RVFV NP were validated on an LFI test to investigate sensitivity and specificity in normal human serum (NHS). Subsequently, these mAbs were implemented in a VFI prototype to investigate the improvement in sensitivity compared to our prototype LFI. Additionally, a preliminary specificity study was performed to evaluate cross-reactivity with diagnostic antigens produced by other known high-priority biothreat agents. The developed VFI diagnostic has great potential for rapid, sensitive, and specific POC detection of RVFV in resource-limited environments.

## MATERIALS AND METHODS

### Monoclonal antibody production

Female 8-week-old BALB/c mice (Charles River Laboratories, Inc., Frederick, MA, USA) and 8-week-old CD-1 mice (Charles River Laboratories Inc.) were immunized intraperitoneally with RVFV rNP (BEI Resources, Manassas, VA, USA) in emulsion with Freund’s complete adjuvant (Millipore Sigma, Billerica, MA, USA). Subsequent immunizations were performed through intraperitoneal injections of RVFV rNP (BEI Resources) emulsified with Freund’s incomplete adjuvant (Millipore Sigma). Blood samples were collected from mice via retro-orbital bleeds. From these bleeds, serum was isolated with the utilization of Microtainer serum separator tubes (Becton, Dickinson and Company, Franklin Lakes, NJ, USA). Antibody titers were assessed via indirect ELISAs in which RVFV rNP (The Native Antigen Company, Kidlington, United Kingdom) was immobilized to the plate. Splenocytes were excised from immunized mice, and hybridoma fusions were performed using a standard protocol (27). Monoclonal antibodies were purified from the hybridoma cell supernatant using standard protein A affinity chromatography.

### Recombinant RVFV nucleoprotein expression and isolation

To obtain additional recombinant antigen for assay development and testing, rNP was produced by the AuCoin laboratory in-house. The sequence for the RVFV NP strain MP12 was determined from GenBank (accession number AF134530.1) (28). The MP12 RVFV NP sequence, along with an N-terminus GS linker and poly-histidine tag, was cloned into the pcDNA3.4 vector by GenScript Biotech. This plasmid was transformed into *Escherichia coli* strain NEB 5α (New England Biolabs, Ipswich, MA, USA). DNA was isolated from the transformed *E. coli* utilizing a ZymoPURE II plasmid maxiprep kit (Zymo Research, Irvine, CA, USA). Purified plasmids were sequence verified prior to mammalian cell transfection. Purified DNA was used to transfect Expi293 (Thermo Fisher Scientific, Waltham, MA, USA) cells using the Thermo Fisher ExpiFectamine 293 transfection kit (Thermo Fisher Scientific). Seven days post-transfection, the rNP was purified using His-Pur Cobalt Resin (Thermo Fisher Scientific). Purified RVFV rNP was verified with Coomassie staining and Western blot reactivity testing. AuCoin lab RVFV rNP was quantified by running an antigen-capture ELISA and generating a standard curve with commercially sourced RVFV NP (The Native Antigen Company). Quantified protein stocks were stored at −80°C until needed.

### Indirect ELISA

Clear 96-well flat bottom polystyrene microtiter plates (Greiner Bio-One, Kremsmünster, Austria) were coated with RVFV rNP (The Native Antigen Company, Kidlington, UK) diluted in 1× Dulbecco’s Phosphate-Buffered Saline (DPBS) (Corning, Corning, NY, USA) at room temperature overnight. Plates were washed three times with a wash buffer consisting of 1× Phosphate-Buffered Saline (PBS) and 0.05% Tween-20 (PBS-T). Following washing, plates were blocked at 37°C for 90 minutes in a blocking buffer consisting of 1× PBS, 0.1% Tween-20, and 0.5% non-fat milk. Upon completion of blocking incubation, plates were dumped and tapped dry on paper towels. Primary antibodies (mouse sera, hybridoma supernatant, or purified mAbs) were diluted in a blocking buffer and added to the plate in appropriate wells. Following the addition of primary antibodies, plates were incubated for 60 minutes at room temperature. Plates were then washed three times with a blocking buffer. The secondary antibody consisted of horseradish peroxidase (HRP)-conjugated goat anti-mouse IgG polyclonal antibodies (Southern Biotech, Birmingham, AL, USA) diluted in a blocking buffer. Purified mAbs were probed with IgG subclass-specific HRP-conjugated goat anti-mouse polyclonal antibodies (Southern Biotech). Secondary labeling was incubated for 60 minutes at room temperature. Plates were washed three times with PBS-T. Room temperature tetramethylbenzidine 2-component peroxidase substrate (SeraCare, Milford, MA, USA) was added to plates and incubated for 30 minutes at room temperature. Following incubation, the substrate reaction was stopped with the addition of 1 M H_3_PO_4_. Plates were read at optical density (OD) 450 nm.

### Western blot

A mini-blotter (Immunetics, Cambridge, MA, USA) adapted Western blot protocol was performed utilizing semidry blotting. This adapted protocol enables the probing of one antigen preparation with multiple antibodies. In brief, RVFV rNP (AuCoin lab) was combined with 6× non-reducing or reducing Laemmli loading buffer (Fisher Scientific, Waltham, MA, USA) to reach a concentration of 1 µg/mL rNP in solution. Reducing samples were incubated at 99°C for 10 minutes to denature proteins. Samples were then pipetted into a 10% sodium dodecyl sulfate (SDS) gel (BioRad Laboratories, Hercules, CA, USA) and separated by running at 140 V for 60 minutes in a 1× tris-glycine SDS buffer. Proteins were then transferred to a nitrocellulose (NC) membrane (BioRad Laboratories). Nitrocellulose was probed with anti-RVFV rNP mAbs at a concentration of 200 ng/mL utilizing the mini-blotter assembly. Following primary, HRP-conjugated goat anti-mouse IgG (Southern Biotech) was used to label the anti-RVFV rNP mAbs. Signal was developed utilizing SuperSignal West Femto Maximum Sensitivity Substrate (Thermo Fisher Scientific, Waltham, MA, USA) and imaged using the ChemiDoc Imaging System (BioRad Laboratories).

### LFI development

Five RVFV mAbs were purified and striped individually at 1 mg/mL in 1× DPBS (Corning) on CN 95 NC membranes (Sartorius, Gottingen, Germany) using a BioDotXYZ3060 (BioDot, Irvine, CA, USA) dispense system. Goat anti-mouse IgG polyclonal antibodies (Southern Biotech) were striped on the control line at a concentration of 1 mg/mL also utilizing the BioDotXYZ3060. Striped NC membranes were adhered to LFI backing cards (DCN Dx, Carlsbad, CA, USA) as well as CSFP203000 wicking pads (Millipore Sigma, Billerica). All purified mAbs were conjugated to 40 nm gold nanoparticles (AuNP) (DCN Dx) utilizing a standard passive adsorption protocol. Each mAb of the RVFV library was tested in every capture and detection position. Positive test samples consisted of RVFV rNP (AuCoin lab) suspended in 1× DPBS (Corning) at a concentration of 100 ng/mL, and negative conditions were buffer only. LFIs were analyzed by eye for test line intensity. Positive test signal/negative test signal was used to compute optimal capture/detection mAb pairs. 1RV4 capture antibody (cAb) and 1RV5 detection antibody (dAb) were selected for LFI development as the pair visually demonstrated the greatest signal intensity.

AuNP-conjugated 1RV5 mAb was sprayed and immobilized on the conjugate pad utilizing an AirJet on the BioDot XY3060 (BioDot). The assay was further optimized for testing human sera. Positive samples consisted of RVFV rNP (AuCoin lab) diluted to various concentrations and spiked into pooled NHS (Innovative Research, Novi, MI, USA), while negative controls contained NHS only. Test and control samples were assayed by pipetting a volume of 40 µL onto the conjugate pad and allowing the prototypes to incubate in running buffer for 15 minutes at room temperature. Gamma-irradiated RVFV ZH501 from infected Vero E6 cells (BEI Resources) was run at a dilution of 1:200. Gamma-irradiated Vero E6 cell lysate (BEI Resources) was also run at a dilution of 1:200. The LFI prototype was optimized to improve positive signal intensity and reduce false positive signals by varying NC types, antibody striping concentrations, conjugate pad composition, and running buffer composition. The final prototype was constructed with UniSart CN 95 NC (Sartorius), CSFP20300 wicking pad, and 8980-grade conjugate pad (Ahlstrom, Helsinki, Finland). Surfactant 10G (Fisher Scientific) and Bovine Serum Albumin (BSA) (Sigma Aldrich) were added to the PBS running buffer to reduce assay background to non-visible as interpreted by three blinded readers.

### VFI membrane and microarray fabrication

VFI membranes were fabricated by immobilizing 1RV4 cAb onto NC membrane discs. The methods used for NC membrane cutting and antibody microarray printing onto these NC membrane discs have been reported previously (23, 29). Briefly, the AutoCAD-drawn membrane pattern was cut into the NC membrane (3.5 mm diameter) using a CO_2_ laser cutter. Using a Nano-Plotter 2.1 (GeSiM, GmbH), a 9-spot microarray pattern was printed onto NC membranes with 1RV4 cAb (5 mg/mL) as the test spots, and goat anti-mouse IgG polyclonal antibodies (Southern Biotech) (1 mg/mL) as the control spots. For cAb optimization, the test spots comprised the top eight spots of the array with one control spot (Fig. 3D). After optimization, the array was developed with six test spots and three control spots arranged in a 2 × 3 grid. Printed VFI membrane discs were then assembled into the fluidic chamber of custom holders with an O-ring, arid silicon support disc, and flangeless fitting prior to assay.

### Optimization of VFI cAb volume and dAb concentration

The microarray was first designed for the optimization of 1RV4 by dispensing 5 mg/mL solution of the cAb with an increasing number of droplets per spot, from 5 to 40. To assess the effect of cAb concentration on VFI sensitivity for RVFV rNP, spiked buffer samples were prepared with 2.5, 0.5, and 0.05 ng/mL of the target antigen (AuCoin lab RVFV rNP). To optimize the AuNP-1RV5 concentration, 3, 6, 9, and 12 pM of 1RV5 dAb were added to the assay buffer without target antigen. Each optimization experiment was performed by passing 5 mL of prepared samples through printed NC membranes.

### LFI and VFI cross-reactivity with known biothreat agents

Target biomarkers of other known Select Agents—*Yersinia pestis* Low calcium responsive V antigen (LcrV) and Fraction 1 antigen (F1), *Francisella tularensis* lipopolysaccharide (LPS), Ebola Zaire Viral Protein-40 (EBOV VP40), and another common viral nucleoprotein (severe acute respiratory syndrome coronavirus 2 nucleoprotein [SARS-CoV-2 NP])—were tested for cross-reactivity. These biomarkers were spiked at a concentration of 100 ng/mL for LFI evaluation and 10 ng/mL for VFI evaluation in NHS samples, then compared with an equal concentration of RVFV rNP and background signal.

### VFI protocol for colorimetric detection of RVFV rNP

A calibration curve was completed by spiking RVFV rNP into assay buffer (0.1 M phosphate buffer containing 0.1% Triton X-100 and 0.5% BSA, pH = 7.2), starting from a 10 µg/mL stock of antigen and serially diluting from 10 to 0.02 ng/mL. Samples were incubated with 9 pM of AuNP-1RV5 for 10 minutes. After incubation, the samples were filtered using a 0.2 µm polyethersulfone (PES) syringe filter and connected to preassembled VFI housings. The sample was flowed at a rate of 0.2 mL/minute using a syringe pump. Control tests were performed with AuNP-1RV5 added to samples without target antigen. To assess VFI performance with the sample matrix, NHS was diluted 10-fold, spiked with 0.31 ng/mL of RVFV rNP, and then serially diluted to 0.02 ng/mL. The assay was then performed by running the spiked serum as aforementioned. After completing the assay, devices were disassembled, and the NC membranes were dried at room temperature for 10 minutes for data analysis consistency. Quantitative analysis of the membranes was done by digitalization with a flatbed scanner for image analysis.

### VFI image processing and statistical analysis

Digitalized membranes were analyzed as described previously (21, 23). Briefly, the signal intensities of microarray spots were measured using custom “VeriFast Image Analyzer” software. Using the unique 3-spot control pattern, test spots are located, and their average intensity is background-subtracted with a nearby location. The limit of detection (LOD) was calculated based on the mean of the blank and three times the standard deviation.

## RESULTS

### Monoclonal antibody production and reactivity

**Fig 1:**
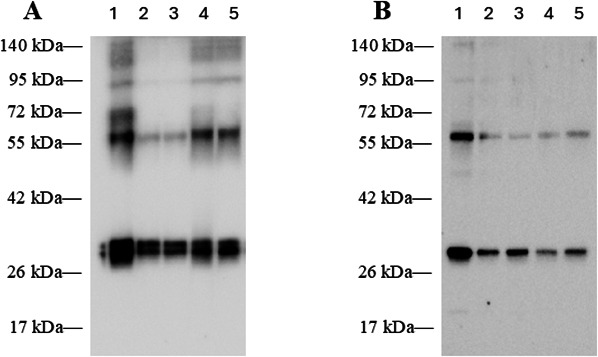
Western blot analysis of RVFV mAb library. Anti-RVFV mAbs 1RV1, 1RV2, 1RV3, 1RV4, and 1RV5 (250 ng/mL) were used in lanes 1–5, respectively. Reactivity to probe non-reduced RVFV rNP (**A**) and reduced RVFV rNP (**B**) is demonstrated. HRP-conjugated goat anti-mouse IgG was used as the detection marker.

Hybridoma cell lines were cloned twice by limiting dilution to ensure both monoclonality and cell line stability. Ultimately, a library of five monoclonal cell lines reactive with RVFV rNP was produced (Table 1). IgG subclass of each mAb was determined by indirect ELISA. Reactivity was demonstrated through Western blot analysis. The RVFV rNP is approximately 28 kDa and readily dimerizes. In the representative blots (Fig. 1), banding is shown in both the non-reduced (Fig. 1A) and reduced (Fig. 1B) Western blots at approximately 28 kDa and 56 kDa, indicating RVFV mAb reactivity with the rNP.

**TABLE 1:** Library of monoclonal antibodies reactive with RVFV NP and corresponding IgG subclass

mAb	Subclass
1RV1	IgG2b
1RV2	IgG2b
1RV3	IgG2b
1RV4	IgG1
1RV5	IgG1

### LFI development

To analyze the prepared library for suitability in a rapid diagnostic, mAbs were first screened in an LFI format. All mAbs were evaluated in the capture and detection positions of the immunoassay. Assay reactivity was first evaluated with RVFV rNP spiked into buffer at a concentration of 100 ng/mL and compared to a non-spiked sample. Both positive test line intensity and negative test line background were visually interpreted by blinded readers. Through this testing, the optimal assay format was developed. The 1RV4 cAb was immobilized on the test line of the assay NC membrane. The 1RV5 dAb was conjugated to gold nanoparticles, and this conjugate (AuNP-1RV5) was sprayed on the LFI conjugate pad.

**Fig 2:**
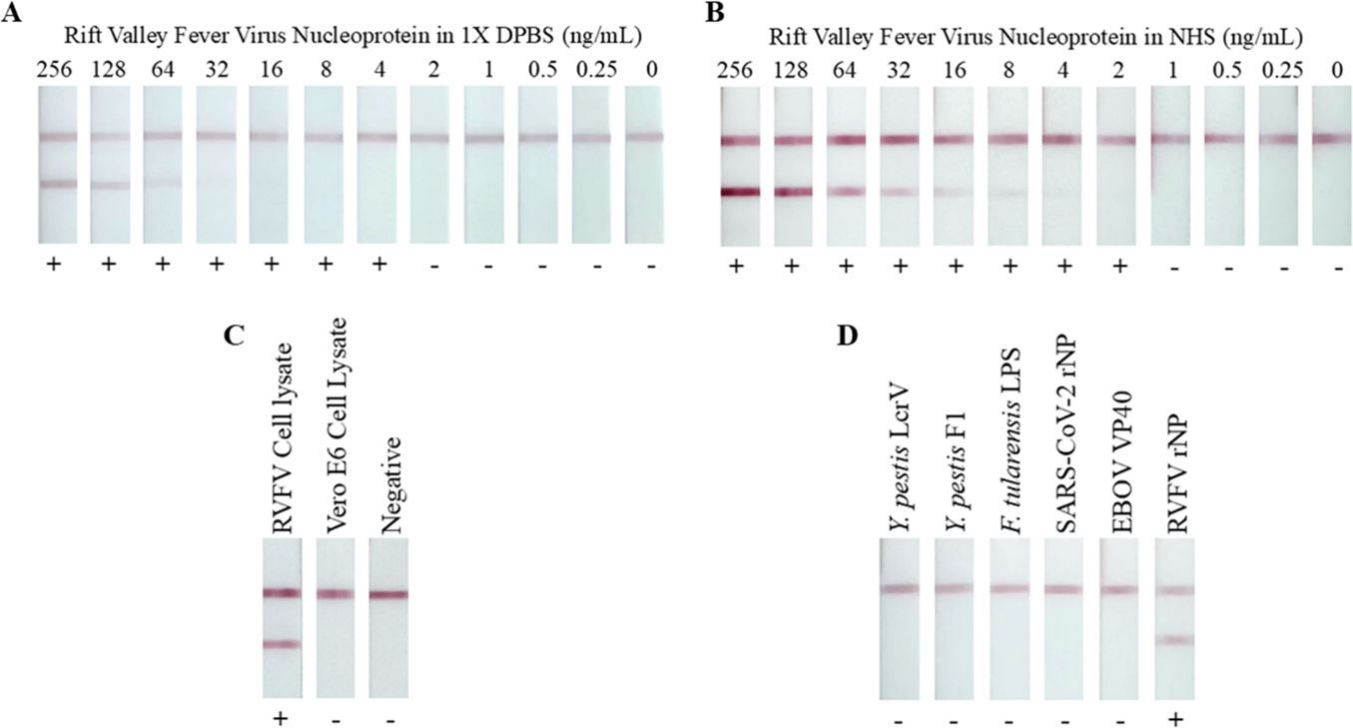
Sensitivity and specificity testing of the RVFV LFI prototype. RVFV rNP suspended in 1× DPBS (**A**) or NHS (**B**) was applied to the sample pad of the LFI prototype at varying concentrations. Presence or absence of a test line, as interpreted by the eye, is depicted as (+) or (−) to indicate the visual result. Gamma-irradiated RVFV lysate suspended in NHS (1:200) was assayed on the RVFV LFI prototype (**C**). Antigen biomarkers from other pathogens were assayed for cross-reactivity (100 ng/mL) in NHS (**D**).

Following pair optimization, the 1RV4/1RV5 LFI prototype was tested with varying concentrations of RVFV rNP suspended in 1× DPBS (Fig. 2A). Solutions of RVFV rNP in 1× DPBS at 256 ng/mL underwent twofold serial dilutions to reach 0.25 ng/mL. These dilutions were tested on the LFI prototype, and a preliminary sensitivity of 4 ng/mL in 1× DPBS buffer was reported. The LFIs were visually assessed by three blinded readers. Due to limitations in the digital camera’s ability to detect faint test lines, reported results reflect assay interpretation with the naked eye. To simulate a clinical sample, RVFV rNP was spiked into NHS (Fig. 2B). The 1RV4/1RV5 LFI prototype yielded improved analytical sensitivity in serum, with reactivity down to 2 ng/mL. To demonstrate that the prototype can recognize RVFV antigen in a more clinically relevant context, gamma-irradiated RVFV lysate (BEI Resources) was tested on the LFI prototype. The dilution of viral lysate in NHS resulted in a positive result (Fig. 2C), confirming the LFI prototype is capable of identifying gamma-irradiated RVFV in spiked human samples. Negative control testing of uninfected Vero E6 cell lysate tested at the same dilution in NHS yielded a negative result.

To demonstrate the specificity of the mAb reagents to RVFV, known diagnostic antigens produced by pathogenic microbes were tested on the LFI. The LFI prototype’s reactivity to *Yersinia pestis* LcrV antigen, *Y. pestis* F1 antigen, *Francisella tularensis* LPS, SARS-CoV-2 nucleoprotein, and ebolavirus Zaire VP40 is shown in Fig. 2D. All antigens in the cross-reactivity panel were spiked at 100 ng/mL in NHS. The 1RV4/1RV5 LFI prototype exhibited no reactivity with these antigens.

### Optimal VFI cAb volume and dAb concentration on VFI

**Fig 3:**
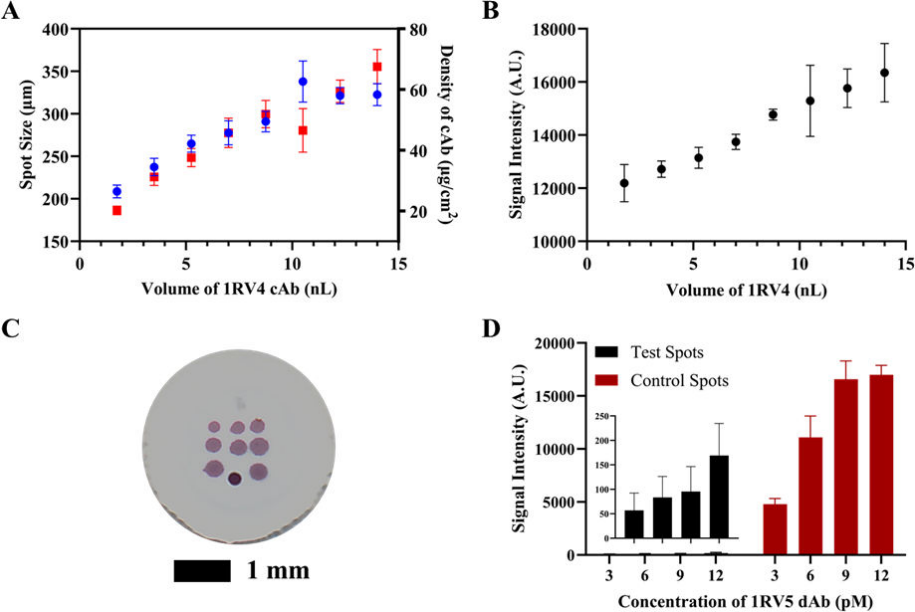
Spot size, density of cAb, and signal intensity reported as functions of the volume of 1RV4 immobilized; VFI membranes were run with 2.5 ng/mL of RVFV rNP in 5 mL of buffer (**A and B**). Image of 1RV4 cAb optimization membrane after assay (**C**). Optimization of dAb concentration involved measuring signal intensity with varying levels of AuNP-1RV5 (**D**).

Building upon the performance of the 1RV4/1RV5 LFI prototype, the same antibody pair was integrated into the VFI platform to further investigate sensitivity. An experiment was first designed to select the optimal volume of 1RV4 cAb because a positive relationship between immobilized cAb and VFI sensitivity was expected until NC saturation had been achieved. Results showed that as the volume of cAb increased from 1.75 to 14 nL (5–40 droplets), the average density of cAb and signal intensity increased from ~20 to 67 µg/cm^2^ and from ~12,000 to 16,000 A.U., respectively (Fig. 3A through C). Interestingly, 10.5 nL of cAb demonstrated the highest spot size and a decreased density of cAb on average. Furthermore, signal intensity became increasingly variable above 8.75 nL of cAb. These findings may suggest that after saturation of the nitrocellulose membrane has been reached, adsorption of additional protein may become inconsistent. Based on these results, 8.75 nL (25 droplets) with a spot size of ~285 µm in diameter and 51 µg/cm^2^ in density of cAb was selected as the optimal volume of 1RV4. Next, the AuNP-1RV5 dAb concentration was optimized. An experiment was performed with an increasing concentration (3, 6, 9, and 12 pM) of dAb in buffer. Results indicate that the control spot signal was saturated at 9 and 12 pM concentrations (Fig. 3D). However, the background signal began to generate at the test spots using 12 pM of dAb. Based on these results, the optimal AuNP-1RV5 concentration was determined to be 9 pM.

### VFI calibration curve and LOD study in NHS matrix

**Fig 4:**
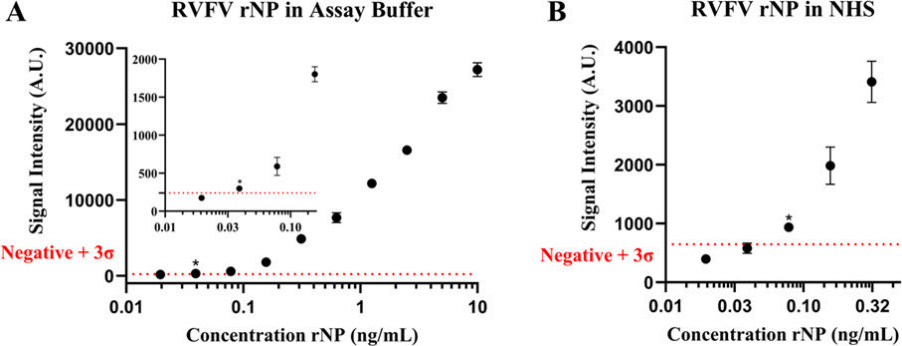
Calibration curve for detection of RVFV rNP in spiked buffer samples (**A**). LOD study for the detection of RVFV rNP in spiked 10% NHS samples (**B**). Background (mean negative + 3σ) signal is shown by red dotted line for control samples, and the LOD is indicated as “*” in each graph.

With optimal VFI components elucidated, the VFI performance was investigated. RVFV rNP was serially diluted from 10.0 to 0.02 ng/mL of antigen in buffer and assayed on the VFI. The observed dose response is depicted in Fig. 4A. Based on the results from this calibration curve, the LOD was determined to be 0.039 ng/mL of rNP using the mean of blank plus three times the standard deviation as background. In subsequent testing, rNP was spiked into NHS in order to simulate the processing of clinical samples collected from RVF patients. Here, a dose response was observed from 0.31 to 0.02 ng/mL of target antigen using 5 mL samples of 10-fold diluted NHS. Results from this experiment indicate a decrease in sensitivity due to an increase in background signal, yielding an LOD of 0.078 ng/mL (Fig. 4B). Due to the dilution factor, this result would correspond to a 0.78 ng/mL concentration of target antigen in the serum alone.

### Cross-reactivity with known biothreat agents

**Fig 5:**
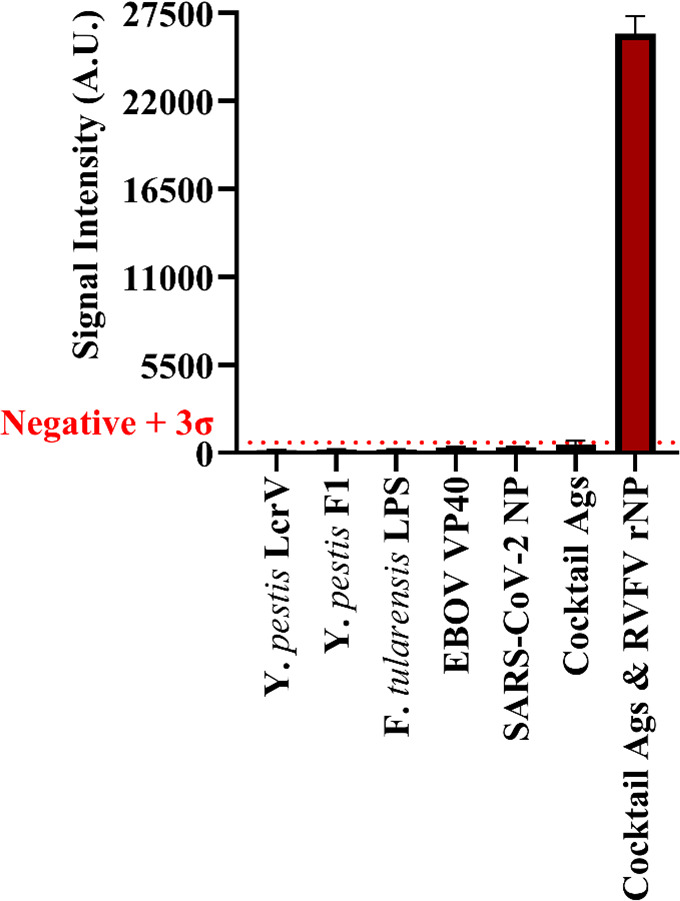
Evaluation of VFI specificity for Rift Valley fever virus nucleoprotein. All antigens were spiked at 10 ng/mL into 10% NHS.

Following LOD testing, the VFI assay was evaluated for specificity. Multiple microbial antigens, as described in Fig. 2D, were run on the VFI prototype. The target biomarkers Y. *pestis* LcrV and F1 antigens, *F. tularensis* LPS, EBOV VP40, and SARS-CoV-2 NP were spiked into 5 mL samples of 10-fold diluted NHS, and the assay was performed. As seen in Fig. 5, the average signals generated by target biomarkers for Select Agents were at or below the background, even with a cocktail of the antigens spiked into a single sample. Furthermore, the assay demonstrated the ability to specifically detect the RVFV rNP within the cocktail of antigens.

## DISCUSSION

Despite being confined to sub-Saharan Africa and the Arabian Peninsula, RVFV remains of great interest to global public health. Due to its potential for economic unrest, risk of epidemic, and ease of spread, both the CDC and WHO have indicated RVFV as a pathogen of great interest. Currently, there exist neither vaccines nor therapeutics to aid in the treatment of RVF. As such, it is of the utmost importance to field sensitive diagnostic tests to aid in the detection and control of RVFV outbreaks. The only licensed diagnostics for RVFV detection are NAATs and ELISAs. Unfortunately, both methods require dedicated laboratory infrastructure and trained personnel—both of which are not always readily available in areas of endemicity. Therefore, there is a need for the development of a rapid, affordable, sensitive test that can be administered at the POC. In this study, we presented data that support the development of an RVFV POC VFI diagnostic device.

Murine immunizations resulted in the isolation of five hybridoma cell lines reactive with RVFV rNP. Following reactivity determination via Western blots, optimal capture/detection pairs for immunoassay development were elucidated. 1RV4 cAb and 1RV5 dAb showed the best signal over the background and were chosen for prototype development. The 1RV4/1RV5 LFI prototype demonstrated increased performance when RVFV rNP was suspended in NHS compared to buffer alone and was able to detect the antigen in NHS at 2 ng/mL. Importantly, the RVFV LFI prototype also readily detected RVFV gamma-irradiated lysate, indicating that the prototype is capable of detecting native viral NP. Additionally, the RVFV LFI prototype showed no cross-reactivity with the small panel of relevant bacterial and viral antigens.

This study builds on the foundational work of Cêtre-Sossah et al., who demonstrated the feasibility of LFIs for detecting RVFV NP (16). While the sensitivity of this LFI was determined to be sufficient for detecting infections in the viremic animals tested, the authors also state that there is evidence of lower-intensity viremia in other animal species, though quantitative data remain difficult to obtain. Furthermore, human RVF cases have mainly been documented in stages of high viremia due to the time in which patients seek medical attention and the diagnosis can be made by qRT-PCR or ELISA (7). This limitation highlights the importance of achieving lower detection limits in POC diagnostics to enable early-stage diagnosis of this vector-borne zoonotic disease in humans and to facilitate appropriate case management.

To address this gap, we optimized the 1RV4/1RV5 antibody pair for integration into the VFI format, further advancing the prototype’s development. The VFI platform introduced key improvements, including increased sample volume capacity and concentration of target signal intensity. These refinements resulted in a 205× increase in analytical sensitivity in buffer and a 25× increase in diluted NHS matrix compared to the LFI prototype. Adjusting for the dilution factor, this suggests an approximate 2.5× increase in sensitivity (i.e., 0.78 ng/mL), highlighting the potential of the VFI as a more sensitive diagnostic tool for RVFV detection.

Additional studies of immunoassays targeting the RVFV NP have reported varying degrees of sensitivity. For example, Van Vuren and Paweska developed a sandwich ELISA with a limit of detection of 11 ng/mL rNP, equivalent to 10^3.2^ tissue culture infectious dose(TCID)₅₀ per mL of whole RVFV antigen, while Fukushi et al. reported a similar ELISA with a detectable limit of 0.8 ng/mL of rNP, corresponding to 78 pfu/mL obtained from cell supernatants (11, 12). More recently, Alharbi et al. introduced a label-free electrochemical aptasensor able to detect rNP with an analytical sensitivity corresponding to 1.5 ng/mL in undiluted serum (17). While it is challenging to directly compare detection limits across studies, both the LFI and VFI presented in this study have analytical sensitivities below those of previously described tests, indicating promise in either assay’s future clinical RVFV diagnostic application. Further testing with clinical samples will be essential to establish clinical sensitivity.

Both the LFI and VFI prototypes affirmed minimal cross-reactivity with panels of biomarkers from other pathogens of interest. The analytical specificity demonstrated with *Y. pestis* F1 and LcrV, *F. tularensis* LPS, and EBOV VP40 shows promise for this integration into a multiplex panel for simultaneous detection of Select Agents. Future studies should focus on validating the assay using clinical samples collected from endemic regions, evaluating field performance in resource-limited settings, optimizing for multiplexed detection of other biothreat agents, and ensuring cost-effectiveness and ease of use for broad adoption and accessibility.

### Conclusion

In this study, we developed the first VFI for the detection of RVFV. This diagnostic device was developed using a sandwich immunoassay to target the RVFV NP, an approach that has been reported and validated by other groups. The reported LOD of 0.78 ng/mL for RVFV rNP in undiluted serum matrix is below the LODs reported from these other groups and provides promise for future studies to test clinical sensitivity. Target analytes of other known biothreats were tested for cross-reactivity in the serum matrix. Signals generated by these analytes were at or below the background, showing minimal cross-reactivity. Specificity will need to be further tested in future studies using samples collected from patients infected with a variety of pathogens. This assay will be integrated into a multiplex panel for the detection of high-priority biothreats on the VeriFAST system for use at the POC (26).
